# Global toxocariasis research trends from 1932 to 2015: a bibliometric analysis

**DOI:** 10.1186/s12961-017-0178-8

**Published:** 2017-02-23

**Authors:** Sa’ed H. Zyoud

**Affiliations:** 1 Division of Clinical and Community Pharmacy, Department of Pharmacy, Faculty of Medicine and Health Sciences, An-Najah National University, Nablus, 44839 Palestine; 2 Poison Control and Drug Information Center (PCDIC), Faculty of Medicine and Health Sciences, An-Najah National University, Nablus, 44839 Palestine

**Keywords:** Toxocariasis, Toxocara, Bibliometric, Scopus, Citations

## Abstract

**Background:**

Toxocariasis is a highly prevalent parasitic disease in the tropical regions of the world, with its impact on public health being typically underestimated. To better recognise the trends and characteristics of toxocariasis research, this study is a bibliometric analysis of the global toxocariasis research.

**Methods:**

Searches were completed on April 5, 2016, using the Scopus database. A search without any language restriction was performed to extract publications dealing with toxocariasis. Terms related to toxocariasis were used to perform a title keyword search.

**Results:**

A total of 2765 publications comprising 11 document types and published between 1932 and 2015 were included in the analysis. Articles were the most popular document form, accounting for 83.62% of all publications, followed by letters (3.80%) and reviews (3.4%). The annual number of research publications increased from 30 in 1980 to 111 in 2015, indicating that the number of publications on toxocariasis has increased slowly over the past 35 years. The United States of America and Japan are the predominant countries of origin, with 303 articles and 207 articles, respectively, followed by Brazil and the United Kingdom, with 180 (6.5%) each. The *h*-index for all the publications was 60. The highest *h*-index were for publications from the United Kingdom (*h*-index value = 43) and the United States (*h*-index value = 39); these two countries were also involved with the highest number of international collaborations, with 27 and 28 countries, respectively.

**Conclusions:**

Developed countries, including the United States, Japan, the United Kingdom, France, Germany and Italy, are the world’s leaders in toxocariasis research, contributing to more than 34% of the total published literature. In addition, developing countries, such as Brazil, Poland, Argentina and India, showed a noticeable increase in published papers on toxocariasis research in recent years. A push for more collaboration is needed to achieve a superior research strategy related to toxocariasis at the global level from the viewpoint of epidemiological data, clinical aspects, medical ecology, molecular aspects and treatment practices associated with toxocariasis.

## Background

Toxocariasis is a highly prevalent parasitic disease in the tropical regions of the world, with its impact on public health being typically underestimated [1–3]. Human toxocariasis is acquired by ingestion of *Toxocara canis* or *Toxocara cati* embryonated eggs present in the soil or on hands and fomites contaminated by the faeces of infected dogs or cats; thus, it is considered to be a widespread zoonotic parasitic disease [1, 4]. *Toxocara* specimens were first illustrated by Werner in 1782; however, the genus was not recognized until 1905 by Stiles [5]. The clinical symptoms of toxocariasis in humans may vary from asymptomatic infection to localized symptoms (ocular and neurological) or severe systemic infection (visceral larva migrans), which is commonly complicated by blood eosinophilia [6–8].

Several studies have concluded that the cost of human toxocariasis is underestimated and understanding of its global impact remains poor because of the inadequacy of clinical awareness and an obvious lack of efficacy of laboratory, clinical and treatment interventions [1, 2, 4, 9–11]. To the author’s knowledge, to date, no bibliometric studies have assessed *Toxocara* and toxocariasis research over time at the global level. The bibliometric technique has already been applied to infectious diseases such as Mayaro [12], Zika [13], Chikungunya [14], leishmaniasis [15], malaria [16, 17], dengue [18, 19], Ebola [20], Middle East respiratory syndrome coronavirus [21] and *Giardia lamblia* [22].

Bibliometric analyses based on measuring the yearly publication output, publication types, source countries with their *h*-index, international collaboration research, source journals with their impact factors (IFs), and citation patterns are widely used by research funders or universities to assess research performance and to shed new light on future research trends. To better recognise the trends and characteristics of toxocariasis research, this study was designed to analyse the global toxocariasis research through bibliometric analysis.

## Methods

The method of this study was derived from those of previous similar studies [17, 23–25]. Searches were completed in April 5, 2016, using the Scopus database. A search without any language restriction was performed to extract publications dealing with toxocariasis. Scopus published by Elsevier is known to be the most common source of data for bibliometric studies in the sciences [17, 25–27]. Compared with other databases, such as PubMed or Web of Knowledge, its records provide more comprehensive coverage [28] of the toxocariasis literature. The terms “toxocar*”, “nematode ophthalmitis”, “visceral larva migrans”, “ocular larva migrans”, “Nematode endophthalmitis”, “dog roundworm” and “cat roundworm” were used as keywords to search titles. These keywords were based on previous review articles [6, 29–31]. The asterisk (*) was applied as a wildcard and enabled the search for variations of key terms. For example, entering “toxocar*” in the Scopus search engine would include the following terms: toxocariasis, toxocara – briefly, any probable word that might start with the seven letters (i.e. ‘toxocar’). Therefore, searching only titles would have resulted in data more related to the field of toxocariasis. No time period restriction was designated in the search concerning the start date, thus all publications prior to December 31, 2015, were included. In this study, a traditional bibliometric technique that included analysis of yearly publication output, languages, publication types, countries with their *h*-index, international collaboration research, source journals with their IFs, citation patterns and institutes was used. Documents published in 2016 or errata were excluded from the analysis. IFs were retrieved from the Journal Citation Reports (JCR 2014) [32]. The values of the *h*-index were extracted from the Scopus database for each country.

### Ethical issues

The analysis in this study is based on a retrospective bibliometric technique; therefore, no ethical approval was required.

### Statistical analysis

**Fig. 1:**
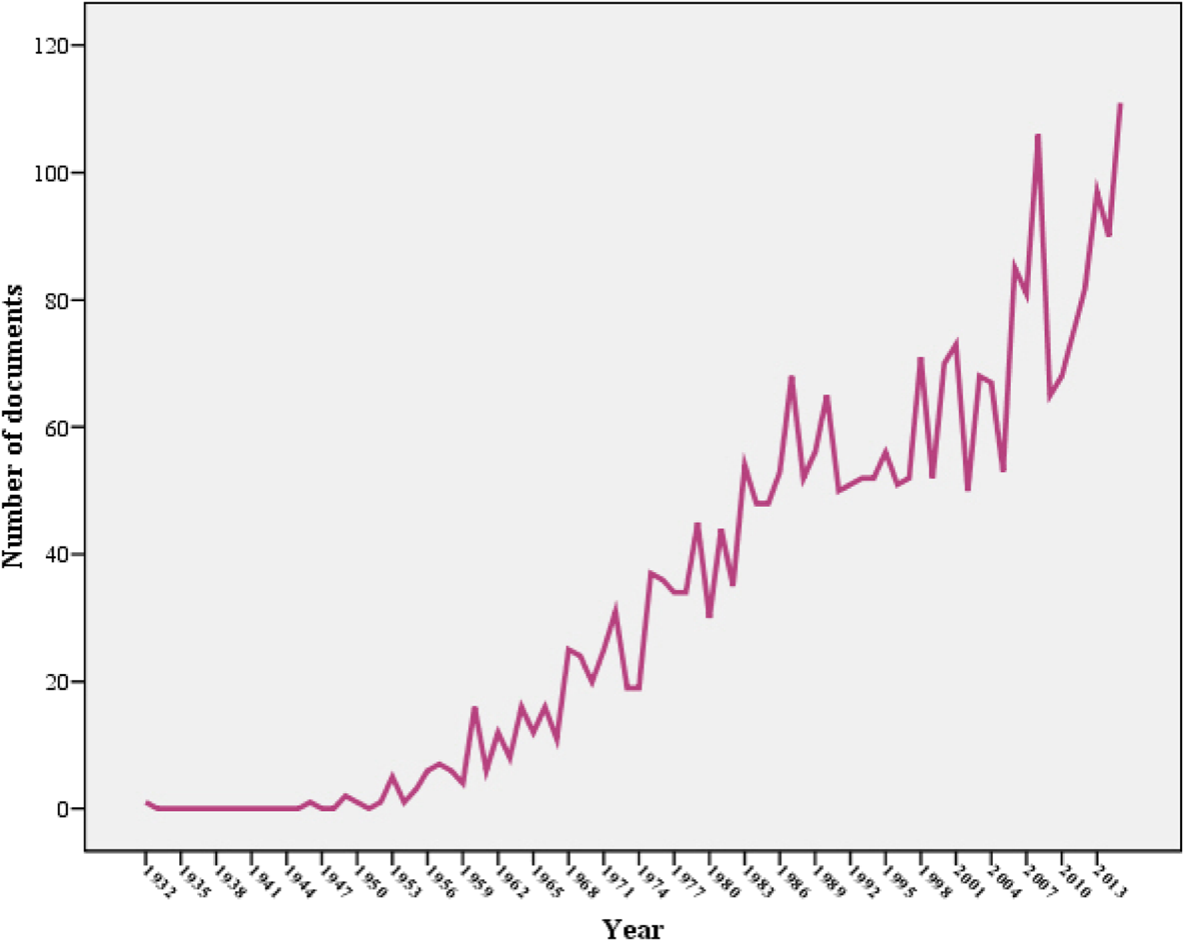
World Scopus publications with toxocariasis during 1932–2015

Microsoft Excel® and version 15 of SPSS® for Windows were used to perform statistical analysis. These software packages were used to generate data on frequency distribution, percentage, sum and average, and to create Fig. 1. Further analysis was introduced to obtain the top ten-ranked prolific countries, most prolific journals, most prolific institutions and most cited papers by using the 1-2-2-4 rule, which is known as the standard competition ranking.

## Results

A total of 2765 publications comprising 11 document types published between 1932 and 2015 were found. Articles were the most popular type of document, accounting for 83.62% of all the publications, followed by letters (3.80%) and reviews (3.4%). Of the 29 different languages identified, English (73.4%), Spanish (4.5%), French (4.5%), German (3.4%) and Polish (3.0%) were predominant. Figure 1 presents the distribution of publications on toxocariasis during the period of 1932–2015. The annual number of research publications increased from 30 in 1980 to 111 in 2015, showing that the number of publications on toxocariasis has increased slowly over the past 35 years.

Concerning the country of publication, 97 countries with publications on toxocariasis were identified. Table 1 shows the top 10 countries in descending order of publication number. The United States of America and Japan were the predominant countries, with 303 and 207 articles, respectively, followed by Brazil and the United Kingdom with 180 (6.5%) each. The *h*-index for all the publications was 60. The highest *h*-index were for publications from the United Kingdom (*h*-index value = 43) and the United States (*h*-index value = 39). The United States and the United Kingdom participated in the highest number of international collaborations, with 28 and 27 countries, respectively. In terms of the rate of publications from international collaborative research to total research for each country, the United Kingdom and Germany (28.9% and 25.3%, respectively) were the most active.

**Table 1:** The 10 most productive countries in toxocariasis research

SCR^a^	Country	Number of documents (%)	*h*-index	Collaborations with foreign countries	The number (%)^b^ of publications with international collaboration^c^
1st	United States	303 (11.0)	39	28	43 (14.2)
2nd	Japan	207 (7.5)	27	19	33 (15.9)
3rd	Brazil	180 (6.5)	23	10	16 (8.9)
3rd	United Kingdom	180 (6.5)	34	27	52 (28.9)
5th	France	115 (4.2)	22	10	14 (12.2)
6th	Germany	79 (2.9)	16	14	20 (25.3)
7th	Poland	78 (2.8)	14	5	11 (14.1)
8th	Italy	61 (2.2)	14	8	10 (16.4)
9th	Argentina	59 (2.1)	15	2	2 (3.4)
10th	India	57 (2.1)	8	2	2 (3.5)

^a^Equal countries have the same ranking number, and then a gap is left in the ranking numbers

^b^Percentage of publications with international collaboration from the total number of publications for each country

^c^“International collaboration” defined as a document with at least two authors from different countries

*SCR* Standard competition ranking

Table 2 lists the 10 journals with the highest number of published documents referring to toxocariasis research from 1932 to 2015, with their IF. *Veterinary Parasitology* published the most documents (96, 3.47%), followed by *Journal of Helminthology* (82), *Revista do Instituto De Medicina Tropical De Sao Paulo* (58) and *Parasitology Research* (57), which had IFs of 2.460, 1.421, 1.007 and 2.098, respectively. The top 10 subject categories worldwide, with greater than 15 publications, are shown in Table 3. *Medicine* comprised 64.4% articles, followed by *Immunology and Microbiology* with 36.1%, and *Veterinary* with 13.1%.

**Table 2:** Top 10 most productive journals, 1932–2015

SCR^a^	Journal	Number of documents (%)	IF
1st	*Veterinary Parasitology*	96 (3.47)	2.460
2nd	*Journal of Helminthology*	82 (2.97)	1.421
3rd	*Revista do Instituto De Medicina Tropical De Sao Paulo*	58 (2.10)	1.007
4th	*Parasitology Research*	57 (2.06)	2.098
5th	*American Journal of Tropical Medicine and Hygiene*	49 (1.77)	2.699
6th	*Journal of Parasitology*	45 (1.63)	1.227
7th	*Transactions of the Royal Society of Tropical Medicine and Hygiene*	37 (1.34)	1.839
7th	*Journal of the Egyptian Society of Parasitology*	37 (1.34)	NA
9th	*Parasite Immunology*	36 (1.30)	2.143
10th	*Parasitology*	35 (1.27)	2.560

^a^Equal journals have the same ranking number, and then a gap is left in the ranking numbers

*SCR* Standard competition ranking, *NA* Not available, *IF* Impact factor

**Table 3:** The top 10 most subject categories in the field of toxocariasis during the study period

SCR	Subject category	Number of documents (%)^a^
1st	Medicine	1780 (64.4)
2nd	Immunology and Microbiology	998 (36.1)
3rd	Veterinary	362 (13.1)
4th	Agricultural and Biological Sciences	340 (12.3)
5th	Biochemistry, Genetics and Molecular Biology	128 (4.6)
6th	Neuroscience	79 (2.9)
7th	Pharmacology, Toxicology and Pharmaceutics	57 (2.1)
8th	Health Professions	28 (1.0)
9th	Environmental Science	17 (0.6)
10th	Chemistry	15 (0.5)

^a^Total of publications exceeds 100% as one paper may fall under different subject categories

*SCR* Standard competition ranking

Table 4 presents the 20 most commonly cited toxocariasis publications between 1932 and 2015 [33–52]. The IFs varied from 1.151 for the 4th most cited paper to 45.217 for the 9th most cited paper. The total number of citations per publication in this table ranged from 108 to 477. The most frequently cited article was published in *Journal of Clinical Investigation* by Del Prete et al. [37] from Italy, which had been cited 477 times.

**Table 4:** The 20 most frequently cited publications related to toxocariasis from 1932 to 2015

SCR	Authors (year of publication)	Title	Source title	Cited by	IF
1st	Del Prete et al. [37]	“Purified protein derivative of Mycobacterium tuberculosis and excretory-secretory antigen(s) of toxocara canis expand in vitro human T cells with stable and opposite (type 1 T helper or type 2 T helper) profile of cytokine production”	*Journal of Clinical Investigation*	477	13.215
2nd	Despommier [38]	“Toxocariasis: Clinical aspects, epidemiology, medical ecology, and molecular aspects”	*Clinical Microbiology Reviews*	364	17.406
3rd	Glickman and Schantz [41]	“Epidemiology and pathogenesis of zoonotic toxocariasis”	*Epidemiologic Reviews*	303	6.667
4th	Magnaval et al. [44]	“Highlights of human toxocariasis”	*Korean Journal of Parasitology*	248	1.151
5th	Beaver et al. [34]	“Chronic eosinophilia due to visceral larva migrans; report of three cases”	*Pediatrics*	243	5.473
6th	De Savigny [35]	“In vitro maintenance of Toxocara canis larvae and a simple method for the production of Toxocara ES antigen for use in serodiagnostic tests for visceral larva migrans”	*Journal of Parasitology*	225	1.227
7th	Schantz [49]	“Toxocara larva migrans now”	*American Journal of Tropical Medicine and Hygiene*	188	2.699
8th	De Savigny et al. [36]	“Toxocariasis: Serological diagnosis by enzyme immunoassay”	*Journal of Clinical Pathology*	164	2.915
9th	Taylor et al. [51]	“The expanded spectrum of toxocara disease”	*The Lancet*	153	45.217
10th	Magnaval et al. [43]	“Application of the Western blotting procedure for the immunodiagnosis of human toxocariasis”	*Parasitology Research*	144	2.098
11th	Overgaauw [46]	“Aspects of toxocara epidemiology: Human toxocarosis”	*Critical Reviews in Microbiology*	134	6.020
12th	Shields [50]	“Ocular toxocariasis. A review”	*Survey of Ophthalmology*	131	3.849
13th	Wilder [52]	“Nematode endophthalmitis”	*Transactions–American Academy of Ophthalmology and Otolaryngology*	130	NA
14th	Jacquier et al. [42]	“Immunodiagnosis of toxocarosis in humans: Evaluation of a new enzyme-linked immunosorbent assay kit”	*Journal of Clinical Microbiology*	119	3.993
15th	Rubinsky-Elefant et al. [48]	“Human toxocariasis: Diagnosis, worldwide seroprevalences and clinical expression of the systemic and ocular forms”	*Annals of Tropical Medicine and Parasitology*	118	1.656
16th	Barriga [33]	“A critical look at the importance, prevalence and control of toxocariasis and the possibilities of immunological control”	*Veterinary Parasitology*	117	2.460
17th	Fisher [39]	“Toxocara cati: An underestimated zoonotic agent”	*Trends in Parasitology*	116	6.204
18th	Glickman et al. [40]	“Evaluation of serodiagnostic tests for visceral larva migrans”	*American Journal of Tropical Medicine and Hygiene*	114	2.699
19th	Overgaauw [47]	“Aspects of Toxocara epidemiology: Toxocarosis in dogs and cats”	*Critical Reviews in Microbiology*	110	6.020
20th	Maizels et al. [45]	“Characterization of surface and excretory-secretory antigens of Toxocara canis infective larvae”	*Parasite Immunology*	108	2.143

*SCR* Standard competition ranking, *NA* Not available, *IF* Impact factor

Table 5 summarizes the top 10 productive institutes. Among them, two were from Brazil, and one each from Slovakia, Ireland, the Netherlands, Germany, Japan, the United States, the United Kingdom, Australia and Sri Lanka. The *Instituto de Medicina Tropical de Sao Paulo* was ranked 1st in institutional productivity with 44 scientific research publications, followed by the *Parasitological Institute of the Slovak Academy of Sciences* with 41 articles and the *Universidade de Sao Paulo* with 40.

**Table 5:** The top 10 productive institutes in toxocariasis research

SCR^a^	Institute	Country	Number of documents (%)
1st	*Instituto de Medicina Tropical de Sao Paulo*	Brazil	44 (1.59)
2nd	*Parasitological Institute of the Slovak Academy of Sciences*	Slovakia	41 (1.48)
3rd	*Universidade de Sao Paulo–USP*	Brazil	40 (1.45)
4th	*Trinity College Dublin*	Ireland	32 (1.16)
5th	*National Institute of Public Health and the Environment*	Netherlands	31 (1.12)
6th	*Tierarztliche Hochschule Hannover*	Germany	25 (0.90)
7th	*University of Miyazaki*	Japan	22 (0.80)
7th	*Cornell University*	United States	22 (0.80)
9th	*London School of Hygiene & Tropical Medicine*	United Kingdom	21 (0.76)
10th	*University of Queensland*	Australia	20 (0.72)
10th	*University of Peradeniya*	Sri Lanka	20 (0.72)

^a^Equal institutes have the same ranking number, and then a gap is left in the ranking numbers

*SCR* Standard competition ranking

## Discussion

The current bibliometric study investigated the global toxocariasis research trends from 1932 to 2015. A bibliometric analysis of the patterns of publication outputs, publication types, journals with their IFs, source countries with their *h*-indexes, international collaboration research, institutional distributions and most-cited articles were conducted.

As shown in the current study, the annual number of research publications increased from 30 in 1980 to 111 in 2015, showing that the number of publications on toxocariasis has increased slowly in the past 35 years. An increase in research output has also been shown in similar research related to infectious diseases such as leishmaniasis [15], malaria [53] and Chagas disease [54]. In a comparison of the number of publications since 1980 concerning toxocariasis, leishmaniasis, malaria and Chagas disease, more rapid growth was observed in the number of papers focusing on leishmaniasis, of which 22,154 publications were published, followed by malaria (36,303 publications) and Chagas disease (5103 publications) compared with toxocariasis, on which 2281 publications were published. From this, it was concluded that, while there was a relative increase in research output in the field of toxocariasis since 1980, there was a higher interest in leishmaniasis, malaria and Chagas disease than in toxocariasis during this time period. This difference in interest between different issues can be attributed to discrepancies in the funding available for different diseases.

Developed countries, including the United States, Japan, the United Kingdom, France, Germany and Italy, are leading countries in toxocariasis research, contributing to more than 34% of the world’s total publications. Possible explanations for these findings may be rapid economic growth or the progress of scientific research systems in these countries. These findings were similar to those reported in earlier bibliometric studies [55–57], which found that the economic growth of a country affected the quantity of research published by its researchers. Developing countries, such as Brazil, Poland, Argentina and India, showed a noticeable increase in published papers on toxocariasis research in recent years, which may have been coincident with a high prevalence of toxocariasis in these countries [31, 58–60]. The United States and the United Kingdom had the highest number of collaborations. Multinational collaboration can help to draw attention to toxocariasis research. Another advantage of this collaboration would be that internationally collaborative publications are usually cited more often than those from individual countries [57, 61].

The most cited article, by Del Prete et al. [37] in 1991, was cited 477 times. This Italian study demonstrated that, in human, the T cell response to *T. canis* comes from the stimulation of T-helper type 2 cell type by the “excretory–secretory” antigens of *T. canis* [37]. The second most cited article was by Despommier [38] in 2003, and looked at epidemiology, clinical and molecular aspects of toxocariasis and the medical ecology associated with the disease.

To date, this is the first bibliometric study to assess the output of peer-reviewed publications on toxocariasis at the global level. Previous bibliometric studies have stated the limitations characteristic of using such an approach [23, 24, 26, 27, 62–64]. First, the publications might not have been included in the analysis if toxocariasis or its related words were not mentioned in their titles, although these terms might have been found in the text. A second limitation was that this study did not include publications on toxocariasis that were in non-indexed journals and thus would not have been available in the Scopus database, such as those published in some Chinese journals.

## Conclusions

This is the first study that investigated the global toxocariasis research trends from 1932 to 2015. The findings indicated that the number of articles published annually increased slowly. Developed countries, including the United States, Japan, the United Kingdom, France, Germany, and Italy, are leading countries in toxocariasis research, contributing to more than 34% of the world’s total publications. In addition, developing countries, such as Brazil, Poland, Argentina and India, demonstrated a noticeable increase in the number of publications related to toxocariasis in recent years. The United States is the world’s leading country in research on toxocariasis and international scientific collaborations on this disease. It is necessary to encourage and support research on toxocariasis in other areas of the world. A push for increased collaboration is needed to achieve a superior research strategy related to toxocariasis at the global level from the viewpoints of epidemiological data, clinical aspects, medical ecology, molecular aspects, and treatment practices associated with toxocariasis. Moreover, the search queries in the current area are biased toward publications in the English language. Therefore, it is important to know that most publications from China may be written in a language other than English, which limits access to these publications for non-Chinese speakers; this may have led to an underestimation of the research activities in non-English countries such as China.

## Abbreviations

IF: impact factor
